# Impact of TNF-α Inhibitors on Body Weight and BMI: A Systematic Review and Meta-Analysis

**DOI:** 10.3389/fphar.2020.00481

**Published:** 2020-04-15

**Authors:** Olivia Patsalos, Bethan Dalton, Jenni Leppanen, Mohammad A. A. Ibrahim, Hubertus Himmerich

**Affiliations:** ^1^ Department of Psychological Medicine, Institute of Psychiatry, Psychology & Neuroscience, King’s College London, London, United Kingdom; ^2^ Department of Clinical Immunological Medicine and Allergy, King’s Health Partners, King’s College Hospital, London, United Kingdom

**Keywords:** TNF-α inhibitor, body mass index, weight, TNF-α blocker, tumor necrosis factor alpha (TNF-α)

## Abstract

**Objective:**

The aim of this systematic review and meta-analysis of longitudinal studies was to ascertain to effects of TNF-α inhibitor therapy on body weight and BMI.

**Methods:**

Three databases (PubMed, OVID, and EMBASE) were systematically searched from inception to August 2018. We identified prospective, retrospective, and randomized controlled studies in adults with immune-mediated inflammatory diseases treated with TNF-α inhibitors based on pre-specified inclusion criteria. A random-effects model was used to estimate standardised mean change (SMCC).

**Results:**

Twenty-six longitudinal studies with a total of 1,245 participants were included in the meta-analysis. We found evidence for a small increase in body weight (SMCC = 0.24, p = .0006, 95% CI [0.10, 0.37]) and in BMI (SMCC = 0.26, p < .0001, 95% CI [0.13, 0.39]). On average, patients gained 0.90kg (SD = 5.13) under infliximab, 2.34kg (D = 5.65) under etanercept and 2.27kg (SD = 4.69) during treatment with adalimumab within the duration of the respective studies (4–104 weeks).

**Conclusion:**

Our results yield further support the for the view that TNF-α inhibitors increase body weight and BMI as a potential side effect. Modulating cytokine signaling could be a future therapeutic mechanism to treat disorders associated with weight changes such as anorexia nervosa.

## Introduction

Tumor necrosis factor alpha (TNF-α) is a proinflammatory cytokine. It has since been found to be produced by various cell types including macrophages, lymphoid cells, endothelial cells, cardiac myocytes, adipose tissue, and brain cells such as microglia and astrocytes. Its receptors are expressed on the surface of every cell type in the human body investigated so far, which reflects TNF-α’s diverse functions. It has been shown to play a key role in immunological defence processes such as inducing fever, inhibiting viral replication during infections, and leading to a permanent growth arrest in cancer (Gaur and Aggarwal, 2003; Braumüller et al., 2013). Its proinflammatory properties have also been implicated in the pathophysiology of autoimmune diseases such as psoriasis (Victor and Gottlieb, 2002), and inflammatory bowel disease (Brynskov et al., 2002). Moreover, altered TNF-α production and signaling have been associated with metabolic disturbances, e.g., obesity (Tzanavari et al., 2010) and cancer cachexia (Batista et al., 2012), and psychiatric and neurological disorders like anorexia nervosa (Vaisman and Hahn, 1991; Plata-Salamán, 1998a; Dalton et al., 2018), Alzheimer’s disease (Swardfager et al., 2010), major depression (Dowlati et al., 2010), and narcolepsy (Himmerich et al., 2006).

TNF-α inhibitors have been studied in context of treatment of patients with several immune-mediated inflammatory diseases such as inflammatory bowel disease (Crohn’s disease, ulcerative colitis), rheumatoid arthritis (RA), ankylosing spondylitis (AS), and psoriasis (NRAS, 2014; Overview; Tobin and Kirby, 2005; Cohen and Sachar, 2017). These medications are either monoclonal antibodies (adalimumab, golimumab, infliximab, certolizumab pegol) or receptor fusion proteins (etanercept) that suppress the physiologic response to TNF-α. Since monoclonal antibodies and fusion proteins need to be produced from living organisms or at least contain components of living organisms, they are referred to as biologic drugs or in short biologics.

Anti-TNF-α antibody therapy has been reported to be associated with an increase in body weight and body mass index (BMI) (Peluso and Palmery, 2016), meaning that TNF-α inhibitors might lead to obesity as a side effect. Obesity in turn is associated with an increased risk of cardiovascular disease (Bray and Bellanger, 2006), metabolic syndrome (Grundy et al., 2004), and mental health problems (Luppino et al., 2010) like depression (Yosaee et al., 2018). In the context of immune-mediated inflammatory diseases (IMIDs), obesity has been associated with more severe disease activity (Liu et al., 2017), inferior quality of life, and suboptimal response to treatment (Singh et al., 2018). Therefore, it would be of value to clinicians to have reliable information about whether TNF-α inhibitors systematically induce weight gain as a side effect and how much weight gain can be expected in a certain time period. If TNF-α inhibitors led to an increase in body weight, blocking TNF-α signaling cold become a novel strategy to treat disorders with severely low weight loss such as cancer cachexia and anorexia nervosa.

While recent a narrative review has mentioned the effects of TNF- α inhibitors on weight (Peluso and Palmery, 2016), neither a systematic review nor a meta-analysis have been performed as yet. Hence, in this report, we will be conducting a systematic review and meta-analysis, evaluating the effects of TNF-α inhibitor treatment on weight and BMI as reported in longitudinal studies.

## Methods

We conducted this meta-analysis according to the Preferred Reporting Items for Systematic Reviews and Meta-Analyses (PRISMA) guidelines (Moher et al., 2009).

### Literature Search

We systematically searched three electronic databases (PubMed, OVID, and EMBASE) from inception until 24^th^ August 2018 using the following search terms: anti-TNF, TNF-α blocker, TNF-alpha blocker, tumor necrosis alpha blocker, tumor necrosis alpha blocker, TNF-α inhibitor, TNF-alpha inhibitor, tumor necrosis alpha inhibitor, tumor necrosis alpha inhibitor, anti-tumor necrosis factor, anti-tumor necrosis factors, golimumab, etanercept, infliximab, adalimumab, certolizumab, in combination with weight, fat mass, body mass, and BMI. These searches were supplemented by internet searches and hand-searches of reference lists of potentially relevant papers and reviews.

### Eligibility Criteria

Searches were limited to studies with adult human participants and those published in English. Any study that assessed weight and/or BMI in the context of anti-TNF-α therapy and reported those measures for at least 2 time points (baseline and follow-up) was eligible for inclusion.

Studies were excluded if: (a) they did not report values for baseline and follow-up, (b) were reporting the use of an experimental biosimilar, (c) stratified results by BMI, or (d) were reporting the effect of BMI or weight on treatment outcome rather than vice versa. Review articles, meta-analyses, conference proceedings/abstracts, book chapters, and unpublished thesis were also not included.

### Study Selection

**Figure 1:**
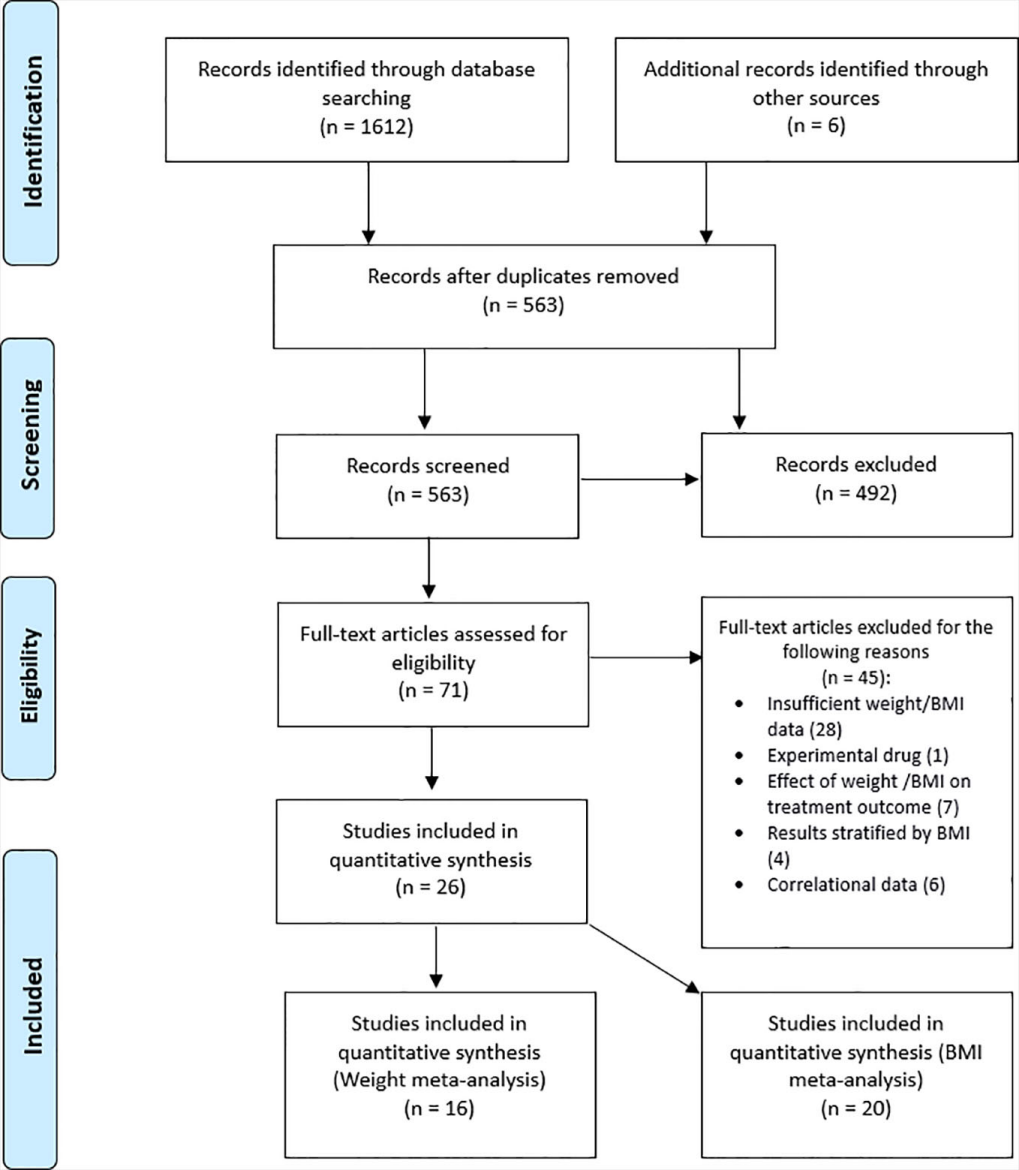
Study selection flowchart of studies reporting weight and/or body mass index (BMI) changes in patients receiving tumor necrosis factor alpha (TNF-α) inhibitors.

The study selection and screening flow chart is presented in **Figure 1**. Titles and abstracts of publications resulting from the search were imported into EndNote and duplicates were removed. Titles and abstracts were screened, and papers deemed highly unlikely to be relevant were disregarded. Full-text versions of the remaining articles were obtained and assessed for eligibility based on our pre-specified inclusion criteria, described above. The entire search process was conducted by two independent reviewers (OP and BD) and disagreements at the final stage were resolved by team consensus. Study quality assessment was performed using the Quality Assessment Tool for Before-After (pre-post) Studies With No Control Group from the National Heart, Lung and Blood Institute (Study Quality Assessment Tools, 2019). Two reviewers, OP and BD independently assessed each study and poor rated studies were discussed among team members.

### Data Extraction and Synthesis

Extracted data from all included studies were compiled into an electronic summary table. Pertinent information such as sample size, means, and standard deviations of weight and/or BMI, and time-frame of treatment was collected. Further parameters of interest such as age, gender, medication type, and clinical diagnosis were also included. As some authors reported their results stratified by medication, each medication was considered separately. If the required data was not reported in the publication, corresponding authors were contacted.

### Statistical Analysis

All statistical analyses were conducted in R Studio (R Core Team, 2015) using the “metafor” package (Viechtbauer, 2010). The primary outcome measure was weight and/or BMI change between baseline and follow-up after treatment commencement with a TNF-α inhibitor. The effect of TNF-α treatment on weight and on BMI was explored in two separate meta-analyses.

The average of the available correlations between baseline and follow-up was the assumedcorrelation for the missing correlations. The following formula was used to calculate standarddeviation if standard error was provided instead: *SD = SEx√N* Standardized mean change with change score standardisation (SMCC) was used to estimate the differences between baseline and follow-up in body weight and BMI (Morris, 2000; Wolff Smith et al., 2009). Cook’s distance (Cook, 1977) was used to explore the presence of influential outliers. Effect size estimates were based on Cohen’s *d* (Cohen, 1992) and were considered small if ≥ 0.20, medium if ≥ 0.50 and large if ≥ 0.80. A random effects model using the *rma* function was used to account for both within-group variability and between-study heterogeneity.

The between-study heterogeneity indices were Cochran’s Q and I^2^. Forest plots were produced as a means of visualization. Meta-regressions were conducted to assess the degree to which the following variables impacted the observed heterogeneity: age, gender, medication type, duration of treatment, and disease. Publication bias was assessed using visual inspection of funnel plots, Begg’s rank correlation of funnel plot asymmetry (Begg and Mazumdar, 1994), and file drawer analysis.

If authors presented data stratified by medication (i.e., infliximab, etanercept, adalimumab, etc), then each medication was considered as a separate dataset in the meta-analysis. The SMCC column represents the effect size estimate of the difference between body weight/BMI baseline and follow-up. Positive effect sizes indicate that body weight/BMI was greater after treatment commencement while positive effect sizes indicate that weight/BMI was lower after TNF-α inhibitor commencement.

## Results

### Study Characteristics

Of the 71 studies that met the eligibility criteria, only 26 studies (N = 1245) reported weight and/or BMI values baseline and follow-up. **Table 1** provides a summary of the study and sample characteristics. None of the studies reported correlational data and only seven authors responded with the requested data. The missing correlations were thus imputed based on the available data (i.e., average of available correlations).

**Table 1:** Characteristics of studies reporting body weight and/or body mass index (BMI) pre- and post-tumour necrosis alpha (TNF-α) inhibitor commencement.

Authors	Study design	Disease	Time-frame (weeks)	N	Medication (dose)	Gender (M)	Age (SD)	SMCC Weight	SMCC BMI	Other meds (N)	Summary
Csontos et al. (2016)	Prospective	IBD	12	40	Adalimumab (160/80/40mg) Infliximab (5mg/kg)	8 (16) 8 (8)	32.3 (14.4) 35.1 (10.5)	-0.73 [-1.18, -0.28] -0.59 [-1.12, -0.06]	-0.71 [-1.16, -0.27] -0.66 [-1.20, -0.12]	5-ASA Azathioprine Steroids	Weight ↑ Lean muscle ↑
De Oliveira et al. (2008)	Prospective	PsO	22	19	Infliximab (5mg/kg)	8 (11)		-0.25 [-0.70, 0.21]			Weight ↑
Derdemezis et al. (2010)	Prospective	RA	26	30	Infliximab (3mg/kg)	30 (0)	51.8 (14.4)	0.42 [00.4, 0.79]		Methotrexate Prednisolone	Weight ↔
Di Renzo et al. (2011)	Prospective	PsO	24	40	Etanercept Infliximab			-0.37 [-0.69, -0.05]	-0.38 [-0.70, -0.06]		Weight ↑ Lean mass ↑ Total fat mass ↑
Dos Santos et al. (2017)	Prospective	CD	26	23	Infliximab (5mg/kg)	12 (11)	42 (12)	-0.60 [-1.04, -0.15]	-0.58 [-1.02, -0.14]		Weight ↑ Lean mass ↑ Total fat mass ↑ Waist circum. ↑
Ehsani et al. (2016)	Prospective	PsO	26	25	Infliximab	7 (18)	36.9 (13.3)		-1.14 [-1.64, -0.64]		BMI ↑
Ersozlu Bozkirli et al. (2014)	Prospective		12	30	Infliximab (5mg/kg)	7 (23)	34.3 (10.2)	-0.28 [-0.64, 0.09]	-0.26 [-0.62, 0.10]		Weight ↔
Esposito et al. (2009)	Retrospective	RA	26	100	Etanercept	34 (68)	43.8	-0.58 [-0.79, -0.37]	-0.54 [-0.75, -0.33]		Weight ↑
Ferraz-Amaro et al. (2011)		RA	52	16					-0.54 [-1.07, -0.02]		BMI ↑
Franchimont et al. (2005)	Prospective	CD	4	20	Infliximab	8 (12)		-0.14 [-0.58, 0.30]	-0.54 [1.07, -0.02]	Prednisolone (8)	Weight ↑
Gisondi et al. (2008)	Retrospective	PsO	26	98	Etanercept (25mg) Infliximab (5mg/kg)		50.2 (11.1) 46.8 (11.2)	-0.28 [-0.54, -0.01] -0.44 [-0.76, -0.11]	-0.23 [-0.49, 0.03] -0.49 [-0.82, -0.16]		Weight ↑ BMI ↑
Kopec-Medrek et al. (2012)	Prospective	RA	8	16	Infliximab (3mg/kg)	16 (0)			-0.26 [-0.76, 0.24]		BMI ↔
Koutroubakis et al. (2009)	Prospective	IBD	14	22	Infliximab (5mg/kg)	8 (14)	38.6		3.33 [2.26, 4.40]	Prednisolone 5-ASA Azathioprine Methotrexate Salazopyrine Antibiotics	BMI ↑
Magiera et al. (2013)	Prospective	RA	53	18	Infliximab (3mg/kg)	18 (0)			-0.17 [-0.64, 0.29]		
Mahe et al. (2014)	Prospective	PsO	52	191	Infliximab (5mg/kg)	60 (131)	46.9 (12.8)		-0.17 [-0.32, -0.03]	Methotrexate (32)	Weight ↔
Marcora et al. (2006)	RCT	RA	24	12	Etanercept	9 (3)	54 (11)	-0.18 [-0.75, 0.39]			Weight ↔
Mathieu et al. (2013)	Prospective	AS	52	49	Adalimumab Infliximab Etanercept	19 (30)	46.9 (12.1)		0.16 [-0.13, 0.44]	Anti-hypertensive (10)	BMI ↔
Metsios et al. (2007)	Prospective	RA	12	20		10 (10)		0.10 [-.34, 0.54]	0.11 [-0.33, 0.55]		Weight ↔ Total body fat ↔ Truncal fat ↑
Parmentier-Decrucq et al. (2009)	Prospective	CD	8	21	Infliximab (5mg/kg)	13(8)	32 (8)		1.49 [0.87, 2.11]	Corticosteroids (30) Methotrexate or Azathioprine (78)	BMI ↑
Popa et al. (2009)	Prospective	RA	26	58	Infliximab (3mg/kg)	42 (16)	56 (11)	0.07 [-0.19, 0.32]	0.00 [-0.26, 0.26]	Corticosteroids Methotrexate Salazopyrine	Weight ↔ BMI ↔
Sandoo et al. (2012)	Prospective	RA	12	23		15 (8)	54 (15)		-0.04 [-0.44, 0.37]		BMI ↔
Saraceno et al. (2008)	Retrospective	PsA	48	305050	Adalimumab (40mg) Infliximab (3mg/kg) Etanercept (50mg)	114 (116)	46.7	-0.53 [-0.91, -0.14] -0.25 [-0.53, 0.03] -0.32 [-0.61, -0.04]	-0.49 [-0.86, -0.11] -0.23 [-0.51, 0.05] -0.29 [-0.57, -0.00]		Weight ↑
Tan et al. (2013)	Retrospective	PsO	48	54 63 61	Adalimumab Infliximab Etanercept	18 (36) 12 (51) 16 (45)	49 (11.7) 49.7 (11.8) 52.3 (11.3)		0.04 [-0.22, 0.31] -0.25 -0.50, 0.01] 0.51 [0.24, 0.78]		Weight ↑ BMI ↑
Toussirot et al. (2014)	Prospective	RA	104	20	Adalimumab (40mg) Infliximab (3-5mg/kg) Etanercept (50mg)	6 (14)	48.6	-0.15 [-0.75, 0.44] -0.48 [-1.68, 0.71] -0.29 [-1.11, 0.53]	-0.11 [-0.70, 0.48] -0.49 [-1.69, 0.70] -0.41, -1.24, 0.42]	Methotrexate (6) Leflunomide (2) Corticosteroids (9)	Weight ↑ Total fat mass ↑
Wiedenmann et al. (2008)	RCT	Cancer	8	2828	Infliximab (3mg/kg) Infliximab (5mg/kg)		63.163.6	0.63 [0.22, 1.03] -4.42 [-5.64, -3.20]	0.34 [-0.04, 0.72] -0.15 [-0.52, 0.22]		Lean body mass ↔
Younis et al. (2013)	Prospective	SpA	16	16	Infliximab		53	-0.07 [-0.57, 0.42]	-0.05 [-0.54, 0.44]		Weight ↔ BMI ↔

IBD, inflammatory bowel disease; RA, rheumatoid arthritis; PsO, psoriasis; PsA, psoriatic arthritis; CD, Crohn’s disease; SpA, spondylarthritis; AS, ankylosing spondylitis.

↑ = increase; ↔ = no change.

Twelve studies reported data for both weight and BMI, while four reported data only for weight, and eight reported data only for BMI. Therefore, the meta-analysis for body weight included a total of 16 studies and the meta-analysis for BMI included 20 studies. With respect to the type of medication used, 20 studies reported data on Infliximab, 8 on Etanercept, 5 on Adalimumab, and 3 did not specify the type of TNF-α inhibitor used. The time-frame from baseline to final follow-up varied considerably, with some studies reporting follow-ups at 4 weeks (Franchimont et al., 2005) and others at 3 years (Toussirot et al., 2014). Within that range, two studies had a follow-up at 8 weeks, 4 at 12 weeks, 1 at 16 weeks, 1 at 22 weeks, 1 at 24 weeks, 6 at 26 weeks, 2 at 48 weeks, 2 at 52 weeks, and 1 at 53 weeks.

**Figure 2:**
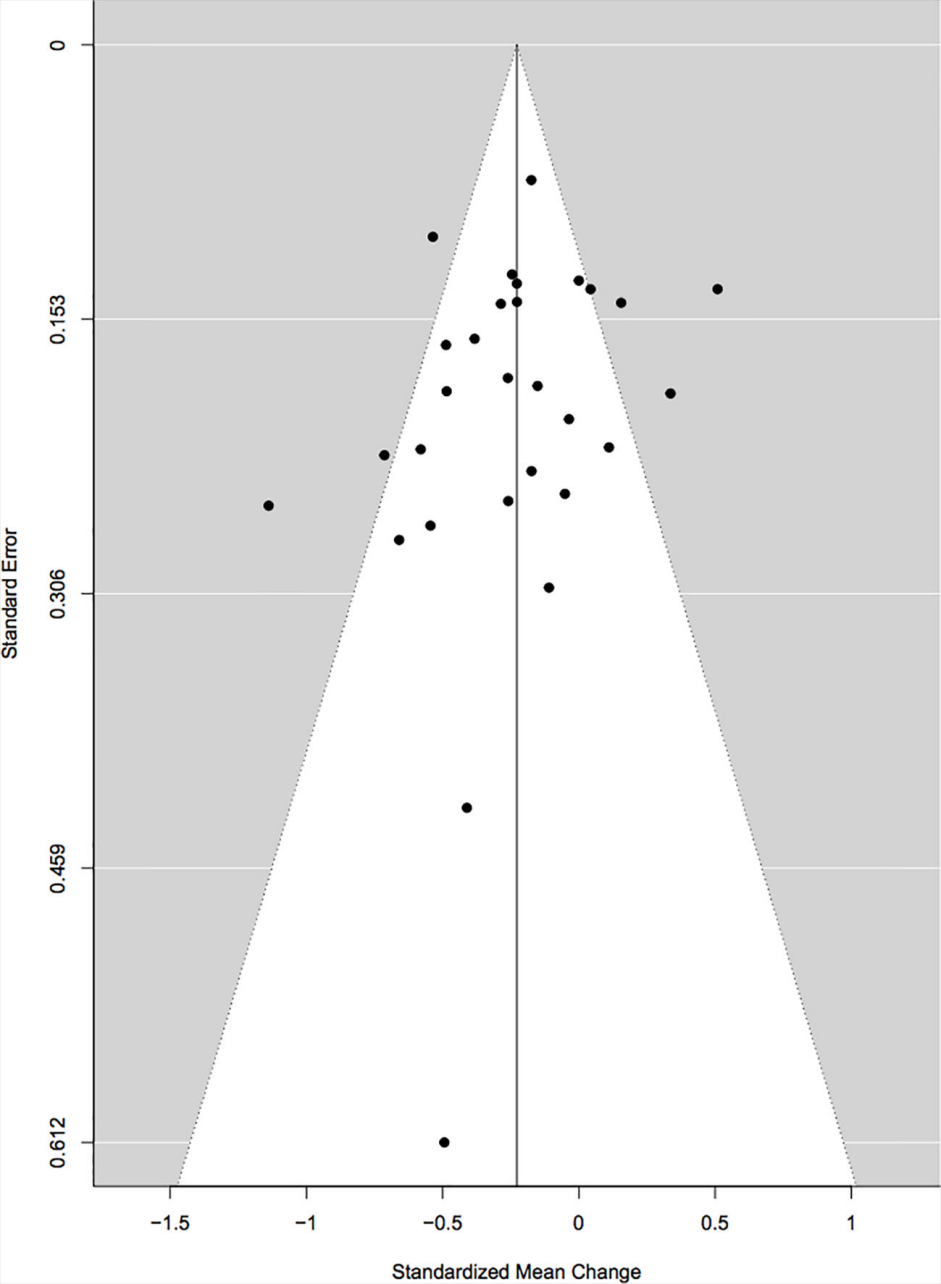
Begg’s rank correlation test for funnel plot asymmetry.

Cook’s distance revealed three outliers (Wiedenmann et al., 2008; Koutroubakis et al., 2009; Parmentier-Decrucq et al., 2009) but upon visual inspection of the forest plots, two were retained (Wiedenmann et al., 2008; Parmentier-Decrucq et al., 2009), and only one was excluded (Koutroubakis et al., 2009). Initially, studies that were rated poor were removed and the analyses were re-run. Since the results with these studies included were not significantly different, it was decided to keep them in the meta-analyses so as to include as many studies as possible. Begg’s rank correlation test for funnel plot asymmetry did not indicate any significant publication bias (τ = −0.18, p = 0.19; **Figure 2**). A further analysis using the Rosenthal approach revealed that the number of null publications needed to reach significant publication bias was 449 (p < 0.0001).

### Impact of TNF-α Inhibitors on Body Weight

**Figure 3:**
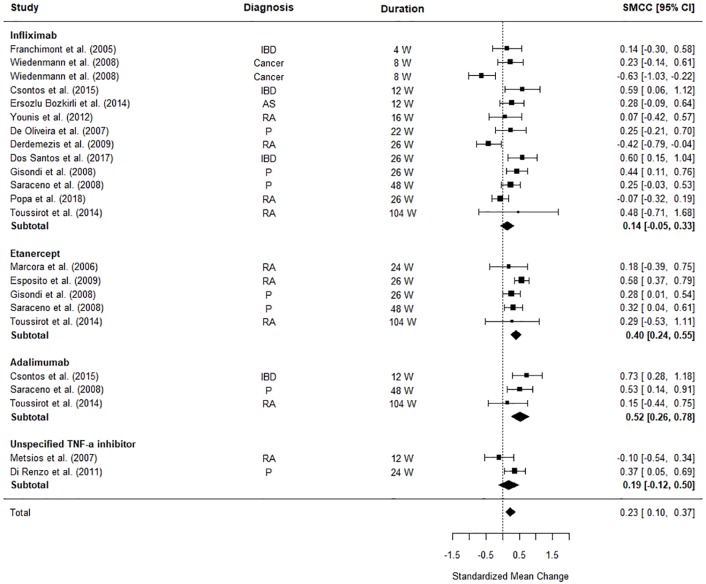
Forest plot of standardised mean change in body weight from 23 datasets (n = 712). Zero indicates no effects whereas points to the right indicate an increase in weight when comparing before and after treatment with a TNF-α inhibitor.

**Figure 3** illustrates the differences between pre and post-TNF-α inhibitor commencement on patients’ weight. The meta-analysis revealed that patients’ weight was significantly increased after TNF-α inhibitor commencement (SMCC = 0.23, z = 3.45, p = .0006, 95% CI [0.10, 0.37]). The weighted pooled mean increase in weight was calculated as 1.49 kg (SD = 5.28) for all TNF- α inhibitors combined, and 0.90 kg (SD = 5.13) for infliximab, 2.27 kg (SD = 4.69) for adalimumab and 2.34 kg (SD = 5.65) for etanercept individually. When each TNF-α inhibitor was considered separately the effects the different medications became clearer. Adalimumab and Etanercept were the main contributors to the significant effect size (SMCC = 0.52, z = 3.92, p < .0001, 95% CI [0.26, 0.78] and SMCC = 0.40, z = 4.96, p < .0001, 95% CI [0.24, 0.55], respectively).

The significant between study heterogeneity (I^2^ = 63.29%, Q = 61.21, p < 0.0001) was further explored using meta-regressions. The meta-regression explained all heterogeneity (Q_moderators_ = 31.38, p < .0001), leaving no significant, unexplained residual heterogeneity (Q_residual_ = 6.34, p = 0.79). The final model was formed of the following moderators: the diagnosis (Psoriasis: Z = 1.90, p = 0.06, 95% CI [-0.02, 1.03], Rheumatoid Arthritis: Z = 2.04, p = 0.45, 95% CI [0.02, 1.08], IBD: Z = 2.53, p = 0.01, 95% CI [0.14, 1.08]), gender (Z = -2.66, p = 0.008, 95% CI [-1.48, -0.23], age (Z = -1.48, p = 0.14, 95% CI [−0.05, 0.01]), and time to follow-up (Z = -0.40, p = 0.69, 95% CI [−0.01, 0.00]). Gender, a diagnosis of rheumatoid arthritis, and a diagnosis of IBD were the main contributors to the study heterogeneity. Studies examining the effect of TNF-α inhibitors on weight in patients with RA or IBD, and studies with samples with fewer females exhibited the largest difference between their baseline weight and their weight post-commencement with a TNF-α inhibitor.

### Impact of TNF-α Inhibitors on BMI

**Figure 4:**
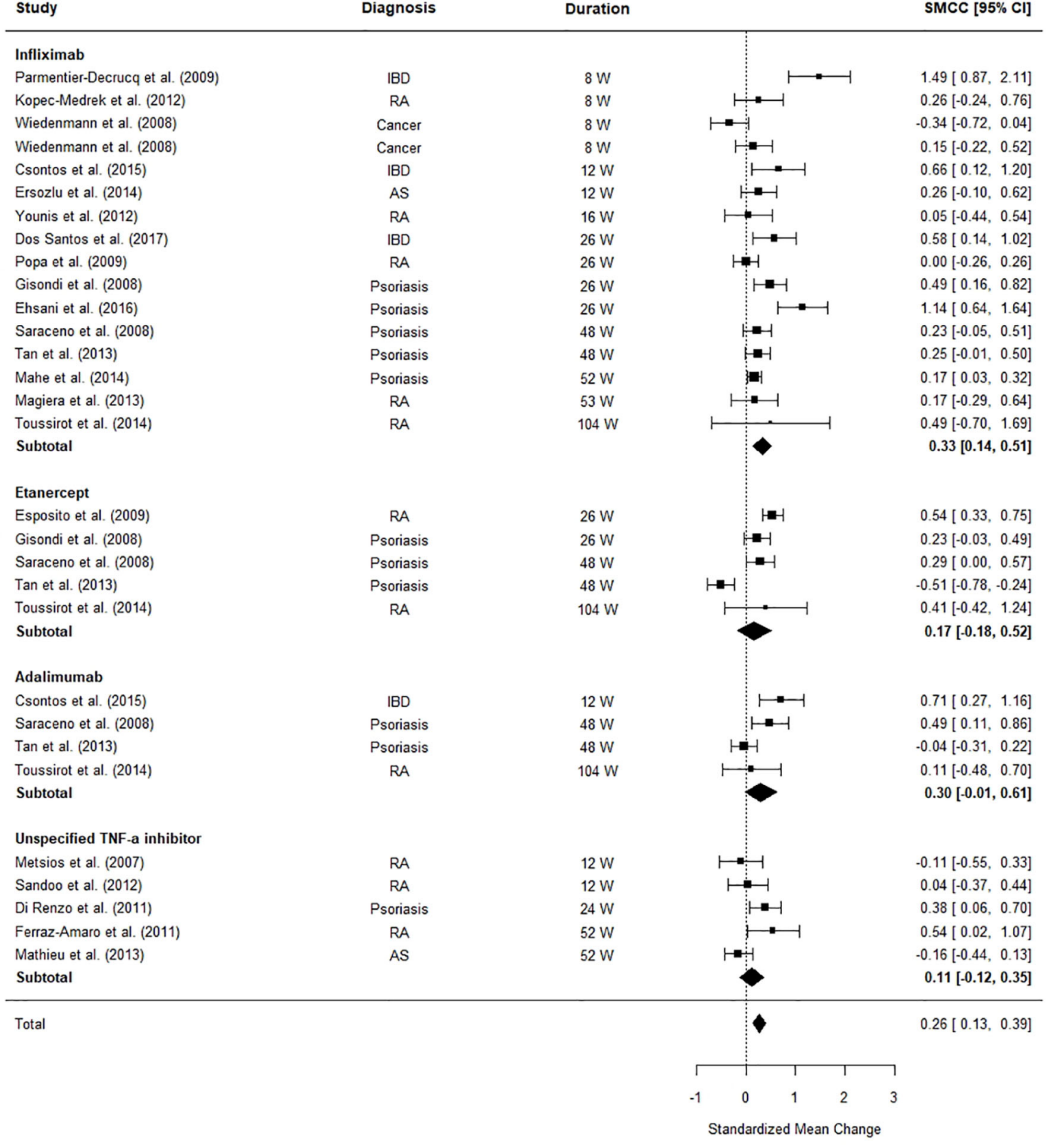
Forest plot of standardised mean change in body mass index (BMI) from 30 datasets (n = 1,156). Zero indicates no effect whereas points to the right indicate an increase in BMI when comparing before and after treatment commencement with a tumor necrosis factor alpha (TNF-α) inhibitor.

**Figure 4** illustrates the differences between patients’ BMI pre and post-TNF-α inhibitor commencement. The meta-analysis revealed that patients’ BMI significantly increased after TNF-α inhibitor commencement (SMCC = 0.26, z = 3.91, p < .0001, 95% CI [0.13, 0.39]). An examination of the subtotals for each medication revealed that infliximab has the most significant effect on patients’ BMI (SMCC = 0.33, z = 3.50, p = .0005, CI [0.14, 0.51]), driving the significant findings, with an average weighted increase of 0.61kg/m^2^ (SD = 2.28).

The significant between study heterogeneity (I^2^ = 76.06%, Q = 109.21, p = < 0.0001) was further explored using meta-regressions. The meta-regression explained a significant portion (91.43%) of the heterogeneity (Q_moderators_ = 49.70, p < 0.0001), however a significant portion of residual heterogeneity was unexplained (Q_residual_ = 29.65, p = 0.01). The final model was formed of the following moderators: clinical diagnosis (Psoriasis: Z = 3.43, p = 0.0006, 95% CI [0.24, 0.87], Rheumatoid Arthritis: Z = 3.41, p = 0.0006, 95% CI [0.27, 0.98], IBD: Z = 1.60, p = 0.10, 95% CI [-0.08, 0.78]), the percentage of females in the sample (Z = 1.11, p = 0.27, 95% CI [-0.34, 1.22], age (Z = -4.89, p < 0.0001, 95% CI [-0.08, -0.03]), and time to follow-up (Z = -0.46, p = 0.64, 95% CI [-0.007, 0.00]. The main model contributors were age, a diagnosis of psoriasis, and a diagnosis of rheumatoid arthritis. These three factors seemed to exhibit the largest effect in terms of difference between patients’ BMI at baseline and follow-up.

## Discussion

### Summary of the Main Results

The aim of the current meta-analysis was to explore the effects of TNF-α inhibitors on body weight and BMI. Seventy-one studies met our screening criteria. However, only 26 of those provided data on baseline and follow-up means and standard deviations of weight or BMI and could thus be included in the meta-analyses.

From the studies included in the meta-analyses, 9 studies reported a significant overall increase in body weight (Franchimont et al., 2005; De Oliveira et al., 2008; Saraceno et al., 2008; Esposito et al., 2009; Di Renzo et al., 2011; Toussirot et al., 2014; Csontos et al., 2016; Dos Santos et al., 2017) and 11 reported a significant increase in BMI (Gisondi et al., 2008; Esposito et al., 2009; Di Renzo et al., 2011; Tan et al., 2013; Mahe et al., 2014; Toussirot et al., 2014; Csontos et al., 2016; Ehsani et al., 2016; Dos Santos et al., 2017). Seven studies reported no significant change in body weight (Marcora et al., 2006; Metsios et al., 2007; Wiedenmann et al., 2008; Popa et al., 2009; Derdemezis et al., 2010; Younis et al., 2013; Ersozlu Bozkirli et al., 2014) and nine reported no significant change in BMI. None of the studies we looked at reported an overall significant loss of either body weight or BMI. Infliximab was the most commonly used TNF-α inhibitor in the studies we’ve reviewed. Interestingly, the effect of infliximab treatment on patients’ weight did not reach statistical significance, whereas infliximab’s effect on patients’ BMI did. Our findings are in agreement with a recent review (Costa et al., 2019) and a recent meta-analysis (Wu et al., 2020) on weight changes in psoriatic patients receiving biologics who also concluded that treatment with a TNF-α inhibitor was associated with a significant increase in body weight. Relatedly and conversely, it is worth noting that a significant portion of studies included here focused on the effect of BMI on TNF- α inhibitor efficacy, and as summarized in this meta-analysis by Dai et al. (2020), the existence of a relationship between weight/BMI and TNF- α is unequivocal.

In our weight moderation analyses, gender seems to be a contributor to heterogeneity. This is in line with some studies that have reported gender differences, with males treated with TNF-α inhibitors being more likely to put on weight (Brown et al., 2012; Tan et al., 2013; Mahe et al., 2014; Csontos et al., 2016). Even though, we could not include disease severity in our model (due to lack of data), it is likely that it could be a contributing factor. For example, Mahe et al. (2014) noted in their study that patients with more severe psoriasis tended to exhibit increased weight gain.

### Mechanisms How TNF-α Inhibitors Might Lead to Weight Pain

TNF-α can lead to weight loss through two distinct mechanisms, namely by influencing the central weight regulation in the brain (Bernstein, 1996; Plata-Salamán, 1998b; Laviano et al., 2003; Bach et al., 2013), and by leading to catabolic processes in the body periphery (Layne and Farmer, 1999; Guttridge et al., 2000). Therefore, TNF-α inhibition might affect both the central as well as the peripheral mechanisms regulating body weight.

**Figure 5:**
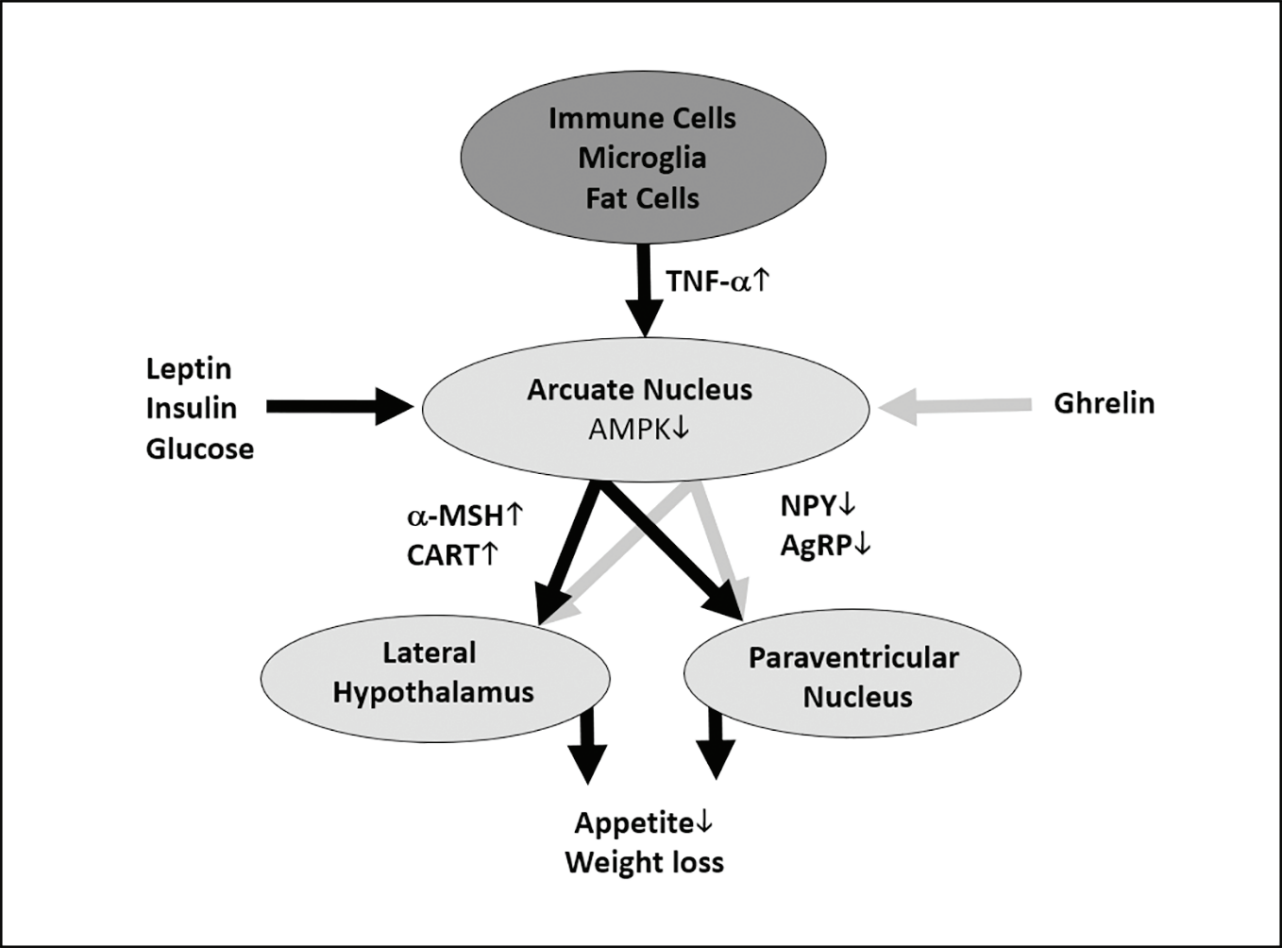
Hypothetical and simplified model of how tumor necrosis factor alpha (TNF-α) could cause anorexigenic effects. TNF-α is released by immune cells, microglia, fat cells, and many other cells (Perskidskiĭ and Barshteĭn, 1992). TNF-α unfolds its anorexigenic effects at the arcuate nucleus of the hypothalamus, which is a central regulator of energy homeostasis, by inducing the production α-melanocyte-stimulating hormone (α-MSH) and cocaine- and amphetamine-regulated transcript (CART) in proopiomelanocortin (POMC)-expressing neurons; additionally, it leads to a decreased production of the orexigenic signals agouti-related protein (AgRP) and neuropeptide Y (NPY) in AgRP-expressing neurons (Romanatto et al., 2007). As TNF-α has been shown to stimulate the intracellular AMP-activated protein kinase (AMPK) (Tse et al., 2017), which integrates orexigenic and anorexigenic signals within the arcuate nucleus (Minokoshi et al., 2004), we hypothesize that this mechanism might play a role in the upregulation of α-MSH and CART and the downregulation of AgRP and NPY. These molecular signals will be conveyed to the lateral hypothalamus and the paraventricular nucleus and thus lead to reduced appetite and weight loss (Claret et al., 2007). However, orexigenic (e.g., ghrelin) and anorexigenic (e.g., glucose, insulin, and leptin) signals from the body periphery modify AMPK activity at the arcuate nucleus (Minokoshi et al., 2004). As mentioned above, this is a simplified figure which neglects important mechanisms influencing the release and the effects of TNF-α. For example, ghrelin can alter TNF-α signaling at cellular level (Himmerich and Sheldrick, 2010). Anorexic signals are depicted as black, orexigenic signals as gray arrows. The dark gray oval represents the entirety of TNF-α-producing cells, the light gray ovals show hypothalamic areas important for appetite and weight regulation.

In the context of cancer cachexia and severe infectious diseases, TNF-α and other proinflammatory cytokines like interleukin (IL)-1β and IL-6 have been shown to induce appetite loss by hypothalamic anorexigenic signaling (Chance et al., 1994; Inui, 1999; Hosoi et al., 2002; Nonaka et al., 2004). Mechanistic studies indicate that this appetite and weight loss is a result of cytokines stimulating the production and release of anorexigenic neuropeptides, such as corticotropin-releasing factor, and inhibiting the signaling with the orexigenic neuropeptide Y (NPY) network (**Figure 5** [(Balt et al., 2011)]). These results are in line with experimental, preclinical and clinical studies showing that IL-1β, IL-6, and TNF-α induce appetite and weight loss when administered peripherally or directly into the brain (Bernstein, 1996; Plata-Salamán, 1998b; Bach et al., 2013). However, it is unclear whether these losses are a result of decreased caloric consumption or some other molecular mechanism involving TNF- α. For example, Metsios et al. (2007) investigated changes in physical activity and protein intake in patients receiving TNF-α inhibitors and found that both increased. Hence, it is possible that the increase in body weight and BMI is a result of increased caloric consumption due to the return of normal appetite.

TNF-α has also been postulated to contribute to muscle loss by inhibiting myoblast differentiation and by mature muscle cell catabolism (Layne and Farmer, 1999; Guttridge et al., 2000). Reid and Li propose that TNF-α stimulates differentiated muscle cell protein loss through its activation of nuclear factor (NF)-κB and subsequent upregulation of ubiquitin/proteasome pathway (Reid and Li, 2001; Patel and Patel, 2017). In line with this, five studies using TNF-α inhibitors reported an increase in lean mass (Briot et al., 2005; Briot et al., 2008; Di Renzo et al., 2011; Csontos et al., 2016; Dos Santos et al., 2017).

It is worth noting, however, that TNF- α has been implicated in the development of obesity and insulin resistance as well. TNF-α is overexpressed in and secreted by adipose tissue of obese animals and humans, and its levels correlate to the degree of adiposity and insulin resistance (Xu et al., 2002; Lorenzo et al., 2008; Tzanavari et al., 2010). Furthermore, it has been shown that a high-fat diet decreases the number of appetite-curbing pro-opiomelanocortin (POMC) neurons by altering their morphology, and that this POMC degradation is mediated through the hypersecretion of TNF-α (Yi et al., 2017). This evidence may at first glance seem contradictory since, broadly speaking, one points to a role of TNF-α as anorexigenic and the other as obesogenic. However, it is plausible given the pleiotropic action of TNF-α that its actions are dose and/or context specific. Even though it is beyond the scope of this review to ascertain the molecular reality of TNF-α actions, it has been previously hypothesised that high elevations of TNF-α contribute to anorexia (Schattner et al., 1992; Berg et al., 1994), whereas in obesity only mild elevations are observed and are linked to insulin resistance (Hotamisligil et al., 1994a; Hotamisligil and Spiegelman, 1994; Hotamisligil et al., 1994b). This position is supported by clinical evidence of naloxone administration, which downregulates TNF-α (Liu et al., 2006). For example, Moore et al. (1981) found that a low dose naloxone (3.2–6.4 mg/day) substantially improved weight gain of anorexia patients without changes in food consumption, whereas large doses (15mg) have been found to be effective in inducing weight loss in obese patients (Wolkowitz et al., 1988). For a more detailed account of the relationship between TNF-α, anorexia, and obesity see Holden and Pakula (Holden and Pakula, 1996).

### Clinical Implications

Our findings suggest that TNF-α blockers could lead to weight gain. According to our analysis, the weighted pooled mean of kilograms gained was 1.49 kg (SD = 5.28). Therefore, weight gain should be considered as a potential side effect of TNF-α inhibitors like golimumab, infliximab, etanercept and adalimumab. However, it is of importance to note that the meta-analyses revealed significant between study heterogeneity and that all estimated standard deviations of change in weight and BMI were large. Our findings mirror the results of some studies where participants lost weight while participants in other studies gained weight. Indeed, some of the primary diseases for which patients were treated with TNF-α blockers, specifically Crohn’s disease and rheumatoid arthritis, are often associated with weight loss (Alastair et al., 2011; Santo et al., 2018). Therefore, weight gain during anti-TNF-α therapy may be interpreted as a restoration of normal body weight. For other disorders, however, such as psoriasis, evidence suggests that obesity is a risk factor, that it aggravates existing psoriasis, and that weight reduction may improve the severity of psoriasis in overweight individuals (Reid and Li, 2001). In these cases, our results of a slight weight gain during therapy with TNF-α blockers suggest that clinical monitoring of patients with regard to weight gain and potentially additional weight-regulating measures such as diet counselling and physical exercise should be considered. Even though our results show only a moderate effect of TNF-α blockers on body weight, in individual cases, weight gain might be excessive and leading to obesity (Tan et al., 2013; Haas et al., 2017). Additionally, our meta-regressions found that other individual differences including gender and age explained some of the variability in weight gain, such that male patients were more likely to gain weight and younger participants showed greater increases in BMI after anti-TNF-α therapy. Therefore, our results support the view that anti-TNF-α medication may lead to a certain amount of weight gain, but further research is still needed to before firm conclusions can be drawn regarding the effect of TNF-α inhibitors on weight gain.

Additionally, modulation of cytokine signaling might be a future mechanism of action for drugs for diseases and disorders associated with weight loss, such as cancer cachexia or anorexia nervosa. In line with this, we have previously suggested that influencing the immune system might be a future pharmacological approach for the treatment of eating disorders (Himmerich and Treasure, 2018; Himmerich et al., 2019). This idea is also supported by findings that TNF-α and IL-6 are elevated in patients with anorexia nervosa (Dalton et al., 2018). Furthermore, administration of a TNF- α inhibitor (infliximab) in a female suffering from both juvenile idiopathic arthritis and anorexia nervosa resulted in an 8% increase in body weight five months after treatment (Barber et al., 2003). The question is, however, whether the amount of weight change that can be expected from therapy with current TNF-α blockers justifies their clinical use in anorexia nervosa. According to our findings, one can expect a mean weight change of 1.49kg (SD = 5.28 or a BMI change of 0.61 kg/m^2^ (SD = 2.28) under TNF-inhibitor treatment. This does not justify anti-TNF-α medication as a sole therapy. However, in addition to one of the established therapies, even a slight extra gain of 0.61 kg/m^2^ BMI might be clinically significant, because it has been shown that weight gain at a scale of 1 kg/m^2^ is associated with improvements in quality of life (Zerwas et al., 2015). As currently available TNF-α blockers can have serious side effects such as tuberculosis, lymphoproliferative disease, lupus-like syndromes, and multiple sclerosis (Lorenzo et al., 2008), it appears unsafe to recommend this type of medication for the treatment of anorexia nervosa. However, they can be kept in mind as a future pharmacological approach or an experimental approach for therapy-resistant and severely affected patients.

### Limitations

The main limitation of this study is that we could only include 26 of the 71 identified studies in the meta-analyses as the required data were not reported and were not available from authors. This meant that potentially informative studies with large samples sizes were not included [e.g., (Wu et al., 2014)]. It also means that among the 26 included studies, there were only two randomized controlled trials (RCT), one testing etanercept versus methotrexate (Marcora et al., 2006), the other comparing infliximab and placebo in patients on gemcitabine treatment (Wiedenmann et al., 2008). Hence, as we had only one placebo-controlled study, we could not execute a meta-analysis on the differences in weight change between treatment with a TNF-α blocker and placebo. Additionally, in some studies patients were also receiving other medications such as methotrexate and prednisone, which have also been shown to influence muscle and adipose tissue and induce weight gain in the short term. Rall et al. (1996) suggested the possibility of methotrexate having a direct anticatabolic effect. Hence, it is possible that the observed weight gain and increased BMI is a result of other concomitant medications, an avenue that would be of benefit if investigated by future studies.

Even though some studies reported such body composition measures (lean mass, fat mass, etc) the available data was insufficient for a meaningful meta-analysis. Given that body weight and BMI are crude measures, it would be beneficial for future studies to ascertain whether the observed gain in those two parameters is a result of gains in fat mass or lean mass, as an increase in fat mass is clinically associated with an increased risk of cardiovascular disease, metabolic syndrome, and depression.

The limited data on potential co-variates such as disease duration, disease severity, smoking, physical activity, and dietary changes did not allow us to carry out more comprehensive meta-regressions where we could explore the effects of TNF-α inhibitors on body weight and BMI in more detail. These are all factors that could differentially contribute to body weight and body composition in general in combination with anti-TNF-α treatment.

Even though Golimumab, Infliximab, Etanercept, and Adalimumab are all inhibitors of TNF-α, there have been reports of differential influence on body weight and body composition. Hence, we were hoping to have sufficient number of studies using the various TNF-α inhibitors to analyse them separately, to explore whether the reported differences remained after being submitted to a meta-analysis. Therefore, we cannot at present compare and contrast the effects of the different TNF-α inhibitors on weight and BMI.

A further limitation is the vastly heterogenous duration of treatment with TNF-α inhibitors. The shortest follow-up was 4 weeks (Franchimont et al., 2005) and the longest was 3 years (Toussirot et al., 2014). Even so, based on our analyses, time to follow-up did not have an effect on weight or BMI. Lastly, the limited data on sample and study characteristics did not allow for a thorough exploration of the significant between-study heterogeneity. Even though we attempted to account for this by performing several meta-regressions, sample and study heterogeneity still poses as a limitation.

## Conclusion

This meta-analysis indicates that administration of TNF-α inhibitors results in an increase in body weight and BMI. Therefore, weight gain could be a side effect TNF-α inhibitors, and TNF-α inhibition could be a potential pharmacological treatment option for the treatment of cancer cachexia or anorexia nervosa. However, it is presently unclear whether the increase in body weight and BMI is as a result of increased fat mass or lean mass. Future studies should additionally consider other contributing variables such as changes in calorie intake, physical activity, appetite, and disease duration and severity.

## Data Availability Statement

Publicly available datasets were analyzed in this study. This data can be found here: Compiled database and R script available on OSF. https://osf.io/fydm.

## Author Contributions

Original idea by HH and BD. OP carried out the review and meta-analysis with guidance from JL and BD. OP drafted the manuscript and the remaining authors contributed with additions and amendments.

## Funding

JL is supported by a Sir Henry Wellcome Postdoctoral fellowship grant (213578/Z/18/Z).

## Conflict of Interest

The authors declare that the research was conducted in the absence of any commercial or financial relationships that could be construed as a potential conflict of interest.
